# Identifying areas of deforestation risk for REDD+ using a species modeling tool

**DOI:** 10.1186/s13021-014-0010-5

**Published:** 2014-11-29

**Authors:** Naikoa Aguilar-Amuchastegui, Juan Carlos Riveros, Jessica L Forrest

**Affiliations:** 1 Forests and Climate Global Initiative, WWF-US, 1250 24th Street NW, Washington 20037, DC, USA; 2 WWF-Peru, Trinidad Moran 853, Lince 14, Lima, Peru; 3 Conservation Science Program, WWF-US, 1250 24th Street NW, Washington 20037, DC, USA

**Keywords:** Carbon, Conservation, Forest, MAXENT, Species habitat modeling, Accessibility

## Abstract

**Background:**

To implement the REDD+ mechanism (Reducing Emissions for Deforestation and Forest Degradation, countries need to prioritize areas to combat future deforestation CO_2_ emissions, identify the drivers of deforestation around which to develop mitigation actions, and quantify and value carbon for financial mechanisms. Each comes with its own methodological challenges, and existing approaches and tools to do so can be costly to implement or require considerable technical knowledge and skill. Here, we present an approach utilizing a machine learning technique known as Maximum Entropy Modeling (Maxent) to identify areas at high deforestation risk in the study area in Madre de Dios, Peru under a business-as-usual scenario in which historic deforestation rates continue. We link deforestation risk area to carbon density values to estimate future carbon emissions. We quantified area deforested and carbon emissions between 2000 and 2009 as the basis of the scenario.

**Results:**

We observed over 80,000 ha of forest cover lost from 2000-2009 (0.21% annual loss), representing over 39 million Mg CO_2_. The rate increased rapidly following the enhancement of the Inter Oceanic Highway in 2005. Accessibility and distance to previous deforestation were strong predictors of deforestation risk, while land use designation was less important. The model performed consistently well (AUC > 0.9), significantly better than random when we compared predicted deforestation risk to observed. If past deforestation rates continue, we estimate that 132,865 ha of forest could be lost by the year 2020, representing over 55 million Mg CO_2_.

**Conclusions:**

Maxent provided a reliable method for identifying areas at high risk of deforestation and the major explanatory variables that could draw attention for mitigation action planning under REDD+. The tool is accessible, replicable and easy to use; all necessary for producing good risk estimates and adapt models after potential landscape change. We propose this approach for developing countries planning to meet requirements under REDD+.

## Background

Deforestation and forest degradation are major sources of greenhouse gas emissions [1]. To address this issue, the 16th and 19th Conference of the Parties (COP-16 & 19) to the United Nations Framework Convention on Climate Change (UNFCCC) agreed on a policy framework for the implementation of the REDD+ mechanism (Reducing Emissions from Deforestation and Forest Degradation) [2],[3]. Under this mechanism, countries need to be able to identify areas at higher risk of future deforestation according to patterns observed in historical deforestation so the information may be used to target areas for mitigation action [3],[4]. These areas of implementation are required to prevent carbon emissions that are likely to occur in the absence of REDD+ actions (ie, provide *additional* benefit to carbon stocks compared with the status quo), and are often defined in practice as high carbon density areas at high risk of loss [4]. Countries also need to identify and characterize the drivers of deforestation and conditions most favorable for deforestation to occur around which to develop mitigation actions, and quantify and value carbon for financial mechanisms [3],[5] (decision 15/19 UNFCCC).

Identification of areas at risk presents methodological challenges. These include: measuring past rates of deforestation and degradation, circumstances and conditions favoring deforestation and degradation in a given place, estimating current carbon stocks, and projecting how human activities and policies can evolve and affect rates of change in the future [6],[7]. Approaches and tools employed in the past to assess areas of future deforestation risk have been successful, but they can also be costly or require considerable statistical knowledge or technical skills to implement [8]-[11]. It is, however, critical that approaches for estimating deforestation risk be relatively easy to employ and accessible so they can be run iteratively to produce a good initial risk prediction and update the model as the human landscape evolves, all within an adaptive management framework [5],[9].

Here, we propose and test a new approach for identifying high deforestation risk areas, appropriate for national and subnational scale REDD+ activity planning and intervention. We test this approach in a subset of the Madre de Dios region of Peru. In recent years, Madre de Dios has undergone a sharp increase in deforestation rates after the enhancement of the Inter Oceanic Highway and subsequent immigration of large numbers of people seeking gold.

We employ a well-established tool and approach historically used for species habitat modeling, the maximum entropy model available in Maxent, to assess the relationship between deforestation and its assumed explanatory variables [12] and to produce a map of deforestation habitat likelihood as a proxy to deforestation risk under a business-as-usual (BAU) scenario. Model performance was assessed using the Receiver Operating Characteristic (ROC) Area Under the Curve (AUC) score generated by Maxent [13]. The AUC Score represents how close the model is to achieve 100% discrimination between presence and absence estimation. The values range from 0-1. If the score is no higher than 0.5 the model being assessed is no better than tossing a coin. We next combined our estimates of deforestation risk with information on past deforestation rates and carbon stocks produced by [14] (which explains the shape of the study area), to estimate potential CO_2_ (CO2) emissions to the year 2020. The results are discussed according to their implications for the region´s carbon policy development as well as for better understanding the impact that a successful REDD+ project could have in the region.

## Results

### Observed historical deforestation

**Figure 1:**
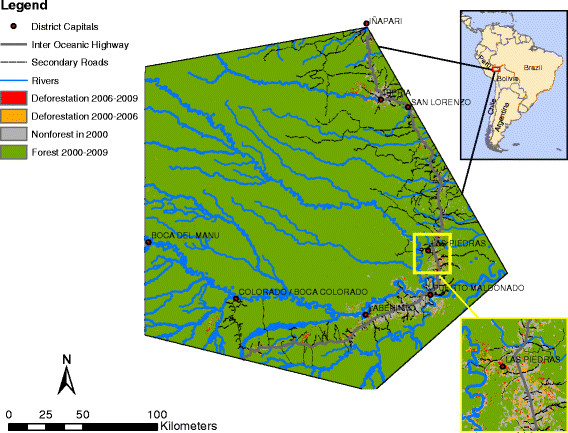
**Deforestation between 2000-2009.** Deforestation has been observed mainly along the main road, in mining areas and close to previously deforested areas.

Over 80,000 ha were lost between 2000 and 2009 inside the study area (≈43,000 ha between 2000-2006 and ≈ 37,000 ha between 2006-2009). This roughly corresponds to 1.9% of the forest area that existed in the year 2000 (4,189,955 ha). Annual deforestation occurred at the rate of *r* = 0.0021 or 0.21% between 2000-2009. Between 2000 and 2006 the deforestation gross rate was 0.17%, whereas between 2006-2009 it almost doubled, reaching 0.30% (Figure 1). The total estimated carbon emissions between 2000 and 2009 were 10,640,000 Mg C or 39,013,330 CO2. 

### Deforestation risk

**Figure 2:**
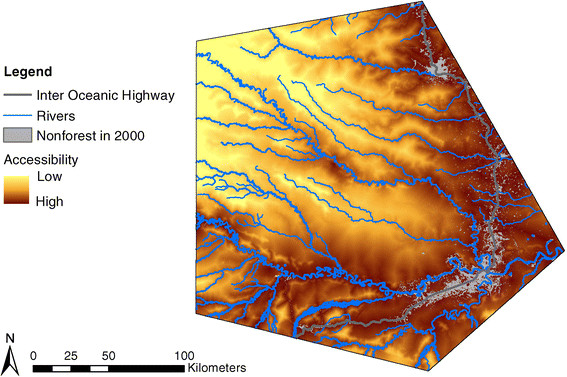
**Accessibility as shown by an Accessibility Index estimated following [**[38]**].** Areas near rivers, roads, and previously deforested land show higher levels of accessibility.

When Maxent was used to evaluate the correlation between observed deforestation and selected explanatory variables for the years 2000-2006, accessibility and distance to previous deforestation emerged as the most important variables, positively correlating with deforestation likelihood (Table 1). Land designation had a relatively minor influence, with mining and public areas (without clear tenure or administrative designation) demonstrating a positive correlation with deforestation risk, and indigenous reservations and protected areas demonstrating a comparatively minor positive correlation (Additional file 1 and Additional file 2). When variables used to prepare the accessibility index (Figure 2) (distance to roads, distance to rivers, land cover, and slope) were used independently in the model, the model performed similarly to when the accessibility layer was used. Sensitivity analyses showed that models produced similar results when land designation was run along with either the accessibility index or distance to deforestation (as estimated with data we had for the period 1990-2000). These results and visual inspection indicate that accessibility and previous deforestation are highly correlated. But, because prior deforestation data was not always available (e.g. due to cloud cover) or its quality unknown (no assessment was carried out), we use only the accessibility index and land designation for the final predictive model, which had good predictive power (AUC = 0.904 and SD = 0.008) (Table 1). 

**Table 1:** Percent contributions of environmental variables to the Maxent model based on 2000-2006 data

**Model**	**Variables**	**Average percent contribution**	**Average AUC score**	**Average SD**
**1**	Accessibility index	32	0.923	0.017
	Distance to previous deforestation	65		
	Land designation	3		
**2**	Accessibility Index	94	0.904	0.008
	Land designation	6		
**3**	Distance to previous deforestation	94	0.920	0.013
	Land designation	6		
**4**	Distance to roads	26	0.904	0.019
	Distance to towns	47		
	Distance to rivers	16		
	Slope	1		
	Land designation	10		
**5**	Accessibility index	95	0.912	0.009
	Land designation	5		

(AUC = Area under curve, SD = Standard deviation).

**Figure 3:**
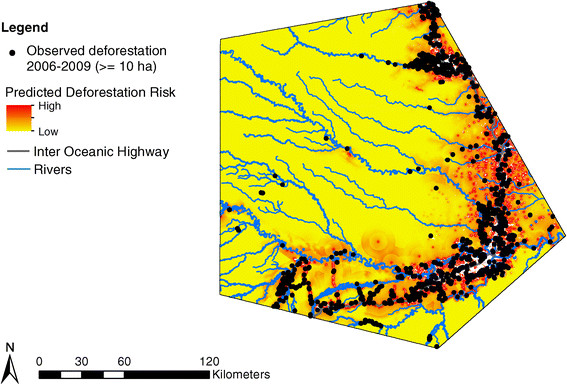
**Soft estimation of future risk of deforestation based on observed deforestation between 2000-2005 overlaid with actual deforestation observed between 2006-2009.** Predicted deforestation risk was based on 2000-2005 explanatory variables and observed deforestation. Nonforest in 2006 is shown in white. Observed deforestation points are based on 2006-2009 data [14]. Note broad scale agreement between predicted and observed locations of deforestation.

**Figure 4:**
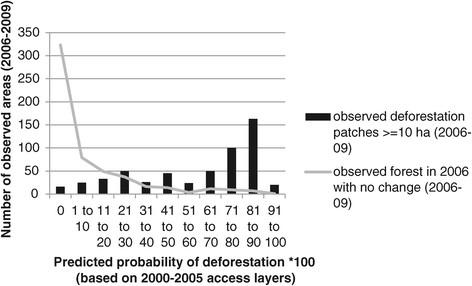
**Comparison between area predicted and area observed deforested 2006-2009.** Black bars indicate that large observed deforestation patches are more likely to be correctly predicted as having a high probability of deforestation (p > 0.59). Observed areas of no forest cover change are more likely to be correctly predicted as having a low probability of deforestation (p < 0.10).

Visual inspection revealed considerable broad scale agreement between deforestation risk and observed deforestation (Figure 3). When we compared the deforestation risk estimates generated in Maxent, based on the 2000-2006 model, with deforestation observed between 2006-2009, we found patches > = 10 ha) that were deforested between 2006-2009 had a significantly higher (Mann-Whitney U test p-value <0.01) average predicted risk of deforestation of 59% with standard deviation (SD) = 29 than areas with no observed forestation from 2006-2009, had a predicted risk of deforestation of 10% (SD =19) (also see Figure 4). These results were obtained even though the deforestation risk estimates were generated based on 2000-2006 explanatory variables, and do not include updated data on new and/or enhanced roads from after 2005. We expect using updated data for the prediction would yieldbetter results. The model still performed significantly well (AUC score >0.5) as exposed both by the AUC scores as well as the risk estimates differences observed for change and no-change locations. 

When we generated a new deforestation risk model based on 2006-2009 observed deforestation and environmental layers, the model was once again deemed acceptable (AUC = 0.912, SD = 0.012). As before, accessibility was the variable with most explanatory power (93% contribution).

**Figure 5:**
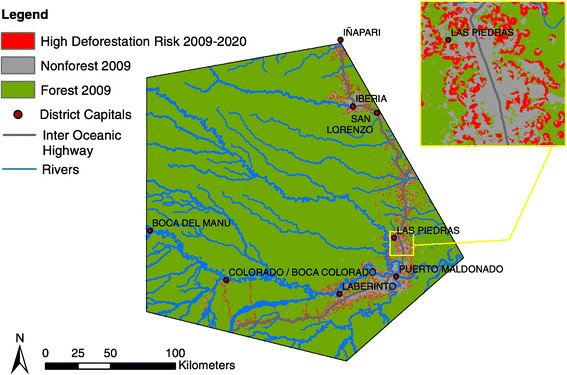
**Hard estimate of forest areas likely to be lost between 2009 and 2020.** Prediction is based on the historical rate observed for the period 2006-2009 (*Hr* = 0.3%) and data on accessibility and land use designation for that same time period.

We identified 132,865 ha where deforestation is most likely to occur by 2020, if 2006-2009 observed rates are maintained, representing 15,013,700 Mg of carbon or 55,050,236 Mg of CO2 (Figure 5). These represent general priority areas for REDD+ mitigation, as well as broad estimates of the amount of CO2 that could be saved as a result of successful REDD+ mitigation actions. 

## Discussion

### Historic deforestation and carbon loss

Deforestation rates in Madre de Dios increased significantly from the early to the late 2000’s. This increase coincided with the enhancement of the Inter Oceanic Highway beginning in 2005, the subsequent influx of gold miners^1^, and the construction of a new secondary, non-planned roads diverting from the highway. Similar to other studies, more accessible areas exhibited higher rates of deforestation [15],[16]. Deforestation rate estimates are affected by a number of technical factors in many cases not well documented, including inconsistent definitions of forest used in the three classifications, details of how data were processed, expert knowledge, etc. Our approach of combining all three sources of deforestation data in which we sought full agreement on deforested areas aimed at only using input deforestation presence points that were 100% valid. However, the net result is likely an underestimation of actual deforestation rates in the study area and should not be used as a reference as it does not constitute an official source. Carbon emissions rates may also be underestimated due to the conservative deforestation rates, the fact that the above-ground biomass estimates we used [14] are low compared with other estimates [17], and because we omit other carbon pools such as below-ground biomass, soil, dead and decaying matter, and harvested wood products. However, above-ground biomass is the most important carbon pool, contributing to the bulk of emissions as a result of deforestation in areas like Madre de Dios [18]. Despite these limitations, and the spatial patterns of deforestation risk produced by this analysis are likely to be useful for mitigation actions targeting on the ground.

### Predictors of deforestation and implications for REDD+ policy development

Accessibility is a key proxy of deforestation risk in Madre de Dios. An increase in accessibility resulting from e.g. newly constructed roads, can increase the deforestation (because of actual cover loss to build the road) as well as increase the risk of additional deforestation to occur from e.g. agriculture and spontaneous settlements along the road [15],[16],[19]. The first significant event in Madre de Dios was the enhancement of the Inter Oceanic highway, which enhanced access and spontaneous settlement by gold miners as a result of an ongoing gold rush into the area. In August 2011, a new bridge, the final link in the ocean to ocean connection provided by the Inter Oceanic Highway, was inaugurated across the Madre de Dios River, which should further improve accessibility to the region and may lead to additional deforestation. Previous to that, vehicles had to be ferried across the river. In both cases, the historic deforestation and potential for future deforestation is largely unplanned, emanating spontaneously from improved access routes [5].

The fact that the human landscape here is rapidly evolving emphasizes the need for periodic updates to access and deforestation data to subsequently update deforestation risk maps and adapt mitigation strategies appropriately. The results obtained in Madre de Dios provide support to arguments of organizations such as the Verified Carbon Standard (VCS) that advise using access data for estimating unplanned deforestation risk, and designing mitigation strategies around core access routes [5].

Land designation showed relatively low explanatory power for deforestation risk when compared with accessibility. While different land designations exhibited different correlations with deforestation rates, we also noted land designations with less restricted use or ease of transformation of use (e.g. protected areas, conservation concessions vs. public non-designated and mining concessions) also appeared to exhibit higher associated risk ([20], Additional file 1 and Additional file 2). It is unclear whether high risk land zones are identified as such because they are close to access routes, or are placed near access routes for the intention of enabling resource extraction and non-conservation uses. Vuohelainen et al. [21] suggest that conservation and ecotourism concessions are among the most effective management types for combatting deforestation in Madre de Dios, while indigenous territories are the least effective. Conservation and ecotourism concessions both allow research, education, ecotourism, and nontimber forest product collection with permit. They differ in that ecotourism concessions pay a fee to the government, but can also exist for profit. Possible reasons for their success at combatting deforestation are that they conduct monitoring and surveillance activities while maintaining good relations with the surrounding communities, all resulting in better governance [21]. It follows from this that conservation and ecotourism concessions (or similarly managed land use zones as we lack data on ecotourism concessions in Madre de Dios) in high or moderate risk areas may be effective at preventing deforestation. However this type of results needs to be carefully assessed as in many cases, such management schemes are implemented in areas of low accessibility (see Figure 1 and Additional file 2), which according to our results should imply a low deforestation risk to begin with. However, implementation in high risk areas could help tackle the intrinsic risk due to location.

### The approach

The Maxent species habitat modeling algorithm was useful for identifying forest areas most likely to be converted and the most important factors associated with deforestation. It enabled us to establish a clear link between observed deforestation rates, distribution of deforestation risk, and the amount of carbon at risk, while achieving a reasonable level of confidence in our prediction. This has clear implications for identifying areas where REDD+ related mitigation actions should be implemented. Other tools and approaches to facilitate deforestation-likelihood modeling for REDD+ are either available at-cost [22] or require a significant amount of user expertise to implement [9],[23],[24]. The strength of this approach is that the tool is freely available, relatively easy to implement, can be applied in an iterative fashion to accommodate better data and update after landscape changes over time (such as new deforestation data deforestation or new infrastructure development), requires presence-only data on deforestation (as opposed to mandatory wall to wall presence/absence data; which is particularly useful in areas such as Madre de Dios that have persistent cloud cover issues), and provides reasonably accurate predictions as required under guidelines such as the VCS (2012).

We note a few limitations and assumptions that are consistent with other modeling approaches of this type. Predictive accuracy of the model is dependent on the availability of updated data on roads and other factors related to deforestation, as well as the accuracy of historic land cover datasets or of input data. It follows that accurate models require periodic data updates to account for new infrastructure changes in land tenure and management and land use developments in particular, as well as for recalibration [5]. Even with the most updated data, the approach is best suited to situations in which future patterns and rates of change do not vary remarkably from past trends. The approach also copes better with explanatory variables of a spatial and local nature (proximate drivers) than policy and socioeconomic factors affecting deforestation agents decision making (ultimate drivers) [19],[25]. For example, the model can better predict change in response to a new infrastructure development than a change in government policy that would affect incentives for people to immigrate to the region. We believe our approach as other similar ones, performs well capturing general patterns deforestation risk, and should not be used to assess risk at the pixel level [26]. Finally, we assumed here that all human causes of deforestation, whether for cropland, development, mining, timber, are a function of the same explanatory variables. The model may perform even better if these were broken out as separate models. It is, however, evident from this analysis that all of these factors are predicted quite well on average using a single statistical relationship.

## Conclusions

We present a simple and practical approach for identifying areas of high deforestation risk that can be considered “additional” areas for REDD+ mitigation actions, and estimating potential forest area loss and carbon emissions under a BAU scenario. The maximum entropy algorithm in Maxent provided an easy to use, freely available, broadly tested algorithm (for species distribution models). In this setting, it provided meaningful assessment of variables related with deforestation risk and meaningful estimates of risk of deforestation. It needs to be indicated, that periodic updating of model fit as per incorporation of new deforestation data and explanatory variables is an ideal exercise as to continuously assess risk behavior as a result of mitigation action implementation as well as to have a constant wach for risk of e.g. leakage. For these reasons, we propose this as a useful and adaptable approach for nations to use for targeting of planning operational strategies to reduce emissions from deforestation and degradation. Our results support guidelines to include accessibility variables when assessing deforestation risk [5]. We note that rapid developments in accessibility in the study area over its 10-year period also emphasize the need for monitoring, reporting, and verification (MRV) schemes that include frequent updates to data and risk projections to ensure that mitigation strategies continue to be effective. Such schemes should identify areas of leakage that might result from REDD+ activities. Additional work is still needed, especially for estimating potential emissions reductions under different development scenarios based on newland designations, opportunity costs, and crop land suitability.

## Methods

To identify areas at high risk of deforestation and carbon loss, we undertook the following four steps: 1) used past land cover data to calculate the historic deforestation rate, 2) used deforestation occurrence data and accessibility-related land cover and land tenure variables in Maxent to prepare a soft prediction, or continuous map, of future deforestation risk; 3) produced a hard prediction of deforestation risk by calculating the expected area to be deforested by the year 2020 (based on past rates of change) and selecting the highest risk pixels from the soft prediction map; and 4) linked areas of high deforestation risk to a forest carbon density map [14] to estimate potential carbon emissions. During this process, we evaluated the accuracy of our modeled predictions by comparing them to observed deforestation.

The study area encompasses 4.3 million ha in the State of Madre de Dios in the Amazon basin of Peru. We selected this area due to its relevance to REDD+ (large forest carbon stocks at high risk of loss, with high biodiversity and important social values), and for the availability of data to complete the study.

### Observed deforestation

#### Deforestation data

Deforestation data were obtained from three different sources. In all cases, data were generated from Landsat TM and ETM+ data acquired between 1999 and 2009. Data sources included: 1) a classification produced by the Carnegie Aerial Observatory (CAO) in collaboration with the Peruvian Ministry of Environment (MINAM) using the CLASlite spectral mixture algorithm tool and automated classifier [14],[27]; 2) a classification produced using a combination of visual and automated classification approaches produced by the Asociación para la Investigación y el Desarrollo Integral” (AIDER)^2^ (AIDER, Internal Report, 2012); and 3) a classification produced by the Madre de Dios REDD Consortium (MdDRC)^3^, whose data were generated using CLASlite in combination with visual interpretation, and with partial groundtruthing performed by crews that regularly work in the area (MdDRC, unpublished data, 2009). All processing approaches generated slightly different outputs. This be can attributed to causes ranging from the specifics of each processing approach to the definition of forest that was used. Reported accuracies for each land cover change map were above 90% [14] (AIDER, internal report, 2012; MdDRC, personal communication, 2012). We decided that rather than choosing a single method, to use only those locations for which deforestation was reported by all three approaches.

For practical purposes, deforestation data were initially divided into 3 periods: 1) 1999-2000 to study the spatial autocorrelation of deforestation (influence of distance to previous deforestation in deforestation likelihood modeling), 2) 2000-2006 for deforestation risk model fitting, and 3) 2006-2009 for 2000-2006 based risk estimate validation. Finally, deforestation data and explanatory variable data from 2006 to 2009 were used to generate soft and hard predictions of deforestation risk, and estimates of potential carbon emissions and emissions reductions under REDD+.

#### Deforestation rate

We estimated the deforestation rate following [28], using the equation:

(1) $$r=\frac{1}{t2-t1}\ln\frac{A2}{A1}$$

Where *r* is the deforestation rate in decimals, *t2-t1* is the difference between the years of the forest cover area assessments (the assessment period), *A1* is the forest area at t1 and *A2* is the corresponding area at *t2*.

We compared our results with those obtained using FAO’s equation [29]. The estimates were very similar (differences approximately 10^−6^) so we kept the rates estimated with equation (1).

### Deforestation risk model

Deforestation results from a set of spatially explicit human behaviors or preferences that occur in response to both local environmental conditions (biophysical and human-made), in addition to larger scale conditions such as market values for resources or government policy [19]. Patterns of deforestation are analogous to species geographic distributions. Species occupy habitat according to their innate preferences for particular habitat types and the existing geography of environmental attributes. Similarly, human-caused deforestation, occurring as a result of the need for cropland, development, or resource extraction, is driven by suitability, access, and other factors that are often geographic in nature. For this reason, we tested the maximum entropy algorithm, Maxent, which is one of the leading algorithms for species habitat modeling [12],[30]-[32], to map deforestation likelihood. Maxent has also been used to map carbon quartiles likelihood (e.g. [33]) and the probability of invasive species infestation [34],[35]. Here, we assumed that future deforestation risk would occur according to past patterns of change, and that the prevailing conditions that facilitated or prevented deforestation would not change significantly in the near future. Our model captured deforestation risk [5] as it pertained to historical patterns of land cover change. We produced both soft and hard deforestation risk estimates. The hard estimates were made up until the year 2020.

### Deforestation occurrence

A random sample of 500 deforestation points observed between 2000 and 2006 in all three deforestation data sources were used as input presence data^4^ for model calibration. Maxent performs well with at least 100 occurrence points when data have been collected without sampling bias [31],[36]. Since a random selection of deforested points was made, the input data was deemed unbiased. Random samples were selected using Hawth’s tools extension for ArcGIS [37].

### Explanatory variables

Explanatory variables selected for the analysis included: distance to previous deforestation that occurred from 1999-2000, land use designation, roads, rivers, land cover type and slope. As a comparison, we also combined roads, rivers, land cover type, and slope into an accessibility index that was calculated using the ArcView 3.0 Accessibility tool [38]. The index calculates how surface type and condition and distance to access networks affect the average speed one can move across the landscape. Average speeds were estimated following [39] and the MdDRC (Additional file 3). The Inter Oceanic Highway was improved in 2005 from dirt to asphalt, which led to increased travel speeds. For this reason, we created two accessibility indexes, one for the 2000-2005 time period (for model fitting, see Figure 2) and one for 2006-2009 (for model testing and projecting future risk).

Land use designations included protected areas, indigenous reservations, forestry concessions, Brazil nut concessions, indigenous communities, ecotourism concessions, and conservation concessions (Additional file 1). All explanatory variables were projected to Universal Transverse Mercator Zone 19 South to reduce distortion errors (see [31]) and resampled to 1 ha cells. Economic and demographic data were not used as they were generally not readily available and the quality of the available data was difficult to assess.

### Maxent model development and performance assessment

We developed a deforestation risk model using 2000-2005 data, running several iterations with different sets of variables to identify the best performing combination of explanatory variables. The different sets of variables tested are described in Table 1. Maxent was run with default settings, using 100 runs with random seeding [31]. We referred to area under curve (AUC) to assess model performance, standard deviation to assess uncertainty, and variable percent contribution for power. Sensitivity tests included removing variables one by one to test the model performance and sensitivity.

In order to assess how well the deforestation risk model performed and the strength of explanatory variables, we also compared areas of observed deforestation and no change in forest cover from 2006-2009 with deforestation risk estimates produced using 2000-2006 data for model fit. Observed deforestation points were identified as the centroid of deforested patches > = 10 ha that occurred between 2006 and 2009. Observed areas with no change in forest cover were identified by randomly generating 500 points over the study area and selecting locations where our deforestation layer indicated forest cover in 2006, and no change from 2006-2009. We produced statistics on the average and standard deviation of predicted deforestation risk estimates for both observed deforestation and unchanged forest cover from 2006-2009. We also produced a histogram to compare observed and predicted deforestation risk (Figure 4).

### Deforestation risk and predicted carbon emissions to 2020

We modeled deforestation risk to 2020 based on 2006-2009 explanatory variables and deforestation occurrence, selecting only those variables identified as most important during model testing and development (described above). We first produced a soft (continuous) prediction of deforestation risk. To produce a hard prediction of forest cover at high risk of loss between 2009 and 2020, we combined the soft prediction with information on rate and amount of predicted loss. We defined the BAU future deforestation rate to be the same as 2006-2009 rates, assuming that the effects of the Inter Oceanic Highway improvement would continue into the future. Using Puyrevad’s deforestation rate equation [28], we calculated the area of forest and forest loss by the year 2020, compared to the baseline year 2009. We produced a map of the areas most likely to be deforested by 2020 by selecting the highest *p* of deforestation values in the Maxent output to where the total area selected matched the area predicted lost, rounding the selection to the nearest whole *p* value.

Finally, we calculated the amount of carbon and CO2 emissions by multiplying the area expected to be lost by the median carbon density of forest (113 Mg/ha) [14]. CO2 emissions were further calculated using the conversion factor -3.67 CO2/Mg C.

## Endnotes

^1^The estimated number of miners increased from 90 to 20,000 in one part of the study area in 2 years (Government of Madre de Dios, personal communication, 2012).

^2^AIDER generates accumulated deforestation data updated on a yearly basis rather than forest/non-forest data

^3^MdDRC is an alliance of local and international NGOs and academic institutions working together to support the development of an MRV system for the State of Madre de Dios in Peru. Founding members include WWF Peru, Government of Madre de Dios (GOREMAD), Conservation International, AIDER, Asociación para la Conservación de la Cuenca Amazónica (ACCA), Universidad Nacional Amazónica de Madre de Dios (UNAMAD), Carnegie Institution for Science, among others.

^4^This restriction was applied due to the fact Maxent has a limit of 1024 KB of memory to run and use of all occurrences made the software crash.

## Abbreviations

AUC: Area under curve

BAU: Business-as-Usual

MdDRC: Madre de Dios REDD Consortium

MINAM: Ministry of the Environment of Peru

REDD+: Reducing emissions from deforestation and degradation with enhancement of carbon stocks

NORAD: Norwegian Agency for Development Cooperation

SD: Standard deviation

## Competing interests

WWF received support for this study from the Government of Norway through a grant administered by the Norwegian Agency for Development Cooperation (NORAD). The views expressed herein are not intended to reflect the policy views of either of these entities or their affiliates.

## Authors’ contributions

NAA and JCR conceived of the research; NAA and JCR gathered data; NAA, JCR and JLF analyzed data; NAA and JLF wrote the manuscript. All authors read and approved the final manuscript.

## Authors’ information

NAA is the Forest Carbon MRV Coordinator of the Forest and Climate Network Initiative at WWF**.** JCR is the Conservation Director of WWF Peru. JLF is a Landscape Ecologist with the Conservation Science Program of WWF US.

## Additional files

## Supplementary Material

Additional file 1: Land designation observed within the study area. Click here for file

Additional file 2: **Deforestation likelihood as it relates to land use designation.** Public areas are those without clear management designation. Click here for file

Additional file 3: **Access friction coefficients used to calibrate the accessibility index [**[38]**] analysis.** Click here for file
