# Supplementary figures and images for: Identifying areas of deforestation risk for REDD+ using a species modeling tool
Source: Carbon Balance Manag. 2014 Nov 29;9:10. doi: 10.1186/s13021-014-0010-5 (PMC4257064; doi:10.1186/s13021-014-0010-5)

Additional File 1. Land designation observed within the study area

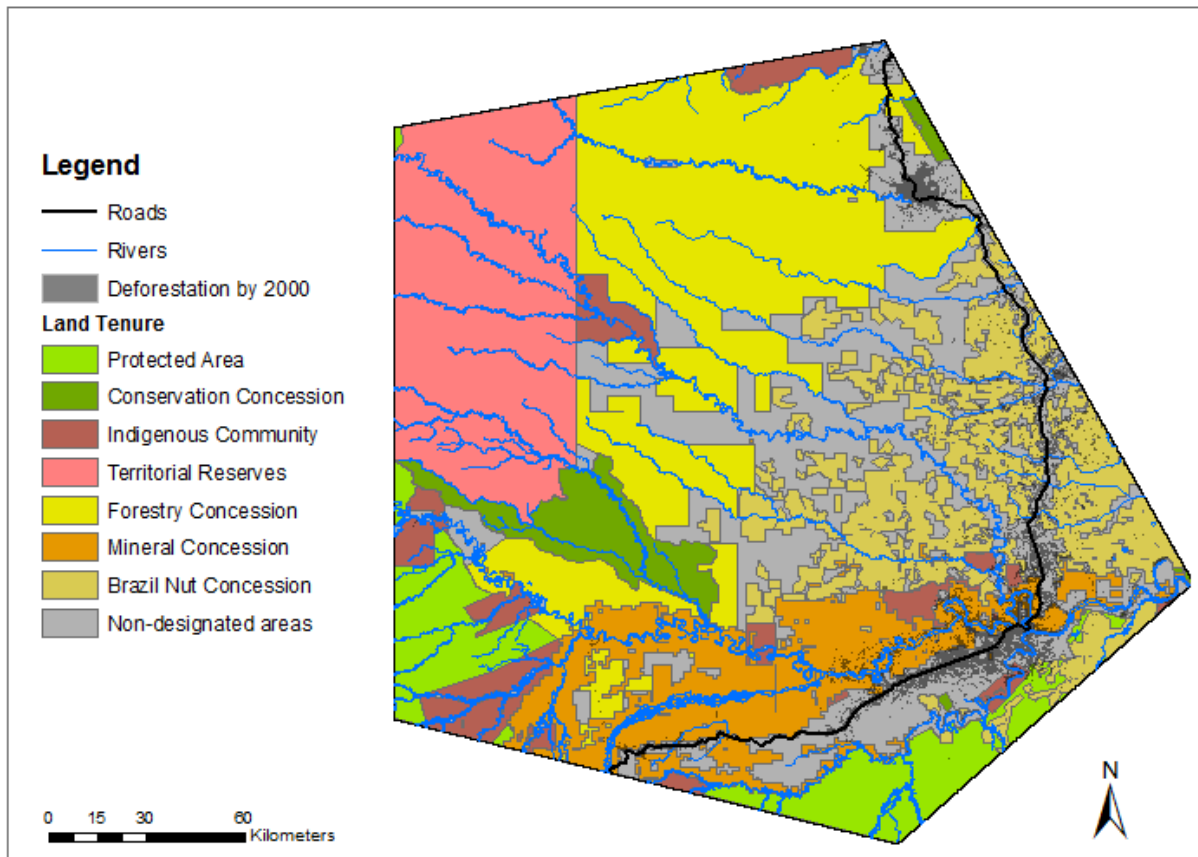

Supplement: Additional file 1: — Land designation observed within the study area. [file s13021-014-0010-5-S1.pdf]
