# Supplementary material for: Identifying areas of deforestation risk for REDD+ using a species modeling tool
Source: Carbon Balance Manag. 2014 Nov 29;9:10. doi: 10.1186/s13021-014-0010-5 (PMC4257064; doi:10.1186/s13021-014-0010-5)

Additional File 2. Deforestation likelihood by land use designation. Mean response of 100 Maxent runs are shown in red, and the mean +/- 1 standard deviation is shown in blue. The graph shows that public areas and mining areas are more strongly correlated to deforestation than other designations, while protected areas, conservation concessions, and indigenous reservations show the least influence on deforestation. (CCNN=Indigenous communities)

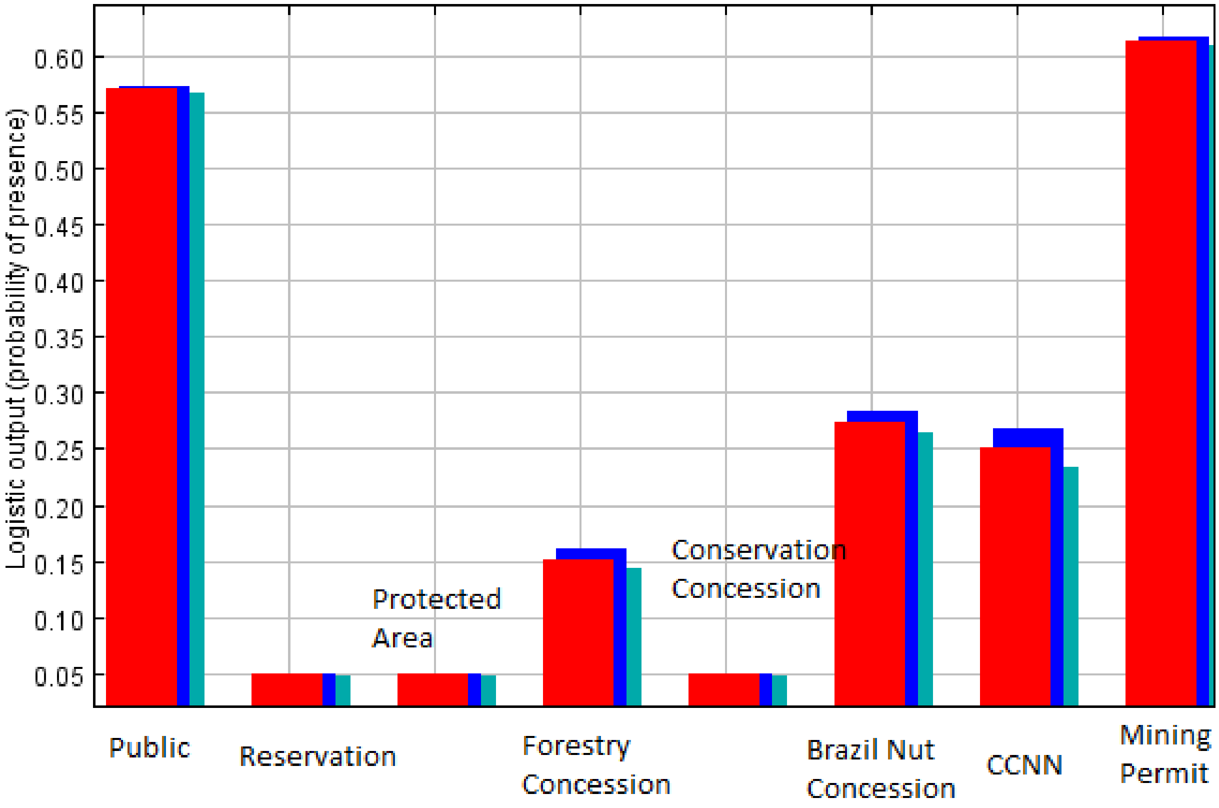

Supplement: Additional file 2: — Deforestation likelihood as it relates to land use designation. Public areas are those without clear management designation. [file s13021-014-0010-5-S2.pdf]
