# Supplementary material for: Identifying areas of deforestation risk for REDD+ using a species modeling tool
Source: Carbon Balance Manag. 2014 Nov 29;9:10. doi: 10.1186/s13021-014-0010-5 (PMC4257064; doi:10.1186/s13021-014-0010-5)
Supplement: Additional file 3: — Access friction coefficients used to calibrate the accessibility index [[38]] analysis. [file s13021-014-0010-5-S3.pdf]

Additional File 3. Friction coefficients used to calibrate the accessibility index analysis. The coefficients were derived following [42]

Surface type	Average speed (km/h)	Friction coefficient (seconds)
**Road –Asphalt**	90	4
**Road Dirt**	30	12
**Road- Track**	10	36
**River (navigable)**	10	36
**Savannah**	4	90
**Swamp Forest**	2	180
**Bamboo Forest**	1	360
**Dense Bamboo Forest**	0.5	720
**Mature Forest**	3	120
**Secondary Forest**	2.5	144
